# Examining trends in the incidence of HIV infection among people with a history of drug use to inform an outbreak investigation and response: A retrospective cohort study

**DOI:** 10.1111/hiv.13712

**Published:** 2024-10-04

**Authors:** Kirsten M. A. Trayner, Norah E. Palmateer, Andrew McAuley, Alan Yeung, Beth L. Cullen, Lesley A. Wallace, Kirsty Roy, Rebecca Metcalfe, Erica Peters, Julie Craik, Daniel Carter, John Campbell, Trina Ritchie, Samantha J. Shepherd, Rory N. Gunson, Sharon J. Hutchinson

**Affiliations:** ^1^ School of Health and Life Sciences Glasgow Caledonian University Glasgow UK; ^2^ Public Health Scotland Glasgow UK; ^3^ Brownlee Centre for Infectious Diseases NHS Greater Glasgow and Clyde Glasgow UK; ^4^ Public Health Protection Unit NHS Greater Glasgow and Clyde Glasgow UK; ^5^ NHS Greater Glasgow and Clyde Addiction Services Glasgow UK; ^6^ West of Scotland Specialist Virology Centre, NHS Greater Glasgow and Clyde Glasgow UK

**Keywords:** HIV incidence, HIV outbreak, people who inject drugs, public health, retrospective cohort study

## Abstract

**Background:**

In the context of an outbreak of HIV among people who inject drugs in Glasgow, Scotland, identified in 2015, our objectives were to: (1) develop epidemiological methods to estimate HIV incidence using data linkage, and (2) examine temporal changes in HIV incidence to inform public health responses.

**Methods:**

This was a retrospective cohort study involving data linkage of laboratory HIV testing data to identify individuals with a history of drug use. Person‐years (PY) and Poisson regression were used to estimate incidence by time period (pre‐outbreak: 2000–2010 and 2011–2013; early outbreak: 2014–2016; ongoing outbreak: 2017–2019).

**Results:**

Among 13 484 individuals tested for HIV, 144 incident HIV infections were observed from 2000 to 2019. Incidence rates increased from pre‐outbreak periods (1.00/1000 PY (95% confidence interval, CI: 0.60–1.65) in 2000–2010 and 1.70/1000 PY (95% CI: 1.14–2.54) in 2011–2013) to 3.02/1000 PY (95% CI: 2.36–3.86) early outbreak (2014–2016) and 2.35 (95% CI 1.74–3.18) during the ongoing outbreak period (2017–2019). Compared with the pre‐outbreak period (2000–2010), the incidence rates were significantly elevated during both the early outbreak (2014–16) (adjusted incidence rate ratio (aIRR) = 2.87, 95% CI: 1.62–5.09, *p* < 0.001) and the ongoing outbreak periods (2017–19) (aIRR = 2.12, 95% CI: 1.16–3.90, *p* = 0.015).

**Conclusions:**

Public health responses helped to curb the rising incidence of HIV infection among people with a history of drug use in Glasgow, but further efforts are needed to reduce it to levels observed prior to the outbreak. Data linkage of routine diagnostic test data to assess and monitor incidence of HIV infection provided enhanced surveillance, which is important to inform outbreak investigations and guide national strategies on elimination of HIV transmission.

## INTRODUCTION

HIV remains a significant public health issue for people who inject drugs (PWID), despite decades of effective prevention through opioid agonist therapy (OAT) and needle and syringe provision (NSP) to reduce injecting risk behaviours associated with transmission, as well as blood‐borne virus (BBV) testing and access to antiretroviral therapy (ART) [1]. Numerous HIV outbreaks among PWID have been documented in high‐income settings with a previously low prevalence of infection [2]. One of the largest and most persistent recent HIV outbreaks has been in Scotland's largest city, Glasgow [2, 3]. Prevalence of HIV among PWID in Glasgow rose more than 10‐fold between 2011 and 2018, associated with homelessness, cocaine injecting and injecting in public places [3, 4]. The public health response to the outbreak has included efforts to increase access to harm reduction services, scale up HIV testing in drug services and prisons, and an enhanced model of HIV care that includes outreach, extensive partner notification and the dispensing of ART alongside OAT [5, 6]. Despite the considerable public health response (full summary of outbreak responses can be viewed in Appendix A), epidemiological evidence pre‐2020 suggested ongoing transmission associated with the outbreak [5].

International studies that estimated HIV incidence in high‐income settings have shown declines among PWID, attributed to the scale‐up of harm reduction and HIV prevention programmes [7, 8]. Incidence (i.e. the rate of new infections) provides an indication of the growth of outbreaks/epidemics and can be used to understand the current rate of transmission and assess the effectiveness of interventions introduced to prevent and control infection [9]. Cohort studies have historically been considered the gold standard approach to measure incidence [10], yet few have been employed to investigate recent HIV outbreaks among PWID [2]. Outbreak investigations typically focus on the number of people that are newly diagnosed (which is influenced by the extent and nature of testing programmes) and can involve case–control or cross‐sectional studies to examine prevalence and risk factors [2].

In contrast to incidence, HIV prevalence measures the overall burden of infection. The ongoing monitoring of HIV prevalence through cross‐sectional bio‐behavioural surveys has provided vital intelligence on the epidemiology of recent HIV outbreaks [2], and this intelligence was pivotal in the early phase of the Glasgow outbreak to demonstrate the rapid spread of infection [3]. However, estimates of incidence also represent important information, particularly during the later outbreak phases where it can be less clear whether a prevalent infection estimated through bio‐behavioural surveys relates to recent or longer‐standing infection. Incidence estimates can also be used to provide intelligence to evaluate the impact of the public health response on reducing HIV transmission associated with the outbreak (Appendix A). Furthermore, in the context of national and international targets to eliminate HIV transmission by 2030 [11, 12], development of methods to estimate HIV incidence among key at‐risk populations is fundamental to monitor progress towards achieving these targets. Specific objectives of this study were to: (1) develop epidemiological methods to estimate incidence utilising retrospective cohort studies and data linkage of multiple administrative sources, and (2) examine temporal changes in HIV incidence among people tested for HIV in Glasgow with evidence of drug use from 2000 to 2019, according to periods of the HIV outbreak (pre‐, early and ongoing outbreak).

## METHODS

### Study design and data sources

We utilised a retrospective cohort study design, including data linkage of laboratory and other healthcare data. Individuals who have accessed healthcare in Scotland receive a Community Health Index (CHI) number [13]. The primary dataset was all laboratory HIV diagnostic tests conducted in National Health Service (NHS) Greater Glasgow and Clyde (NHS GGC) (the administrative location of the HIV outbreak) between 2000 and 2019, obtained from the NHS West of Scotland Specialist Virology Centre. Data were received relating to CHI, sex, date of birth, specimen date of HIV test and HIV test result. Risk factor information (i.e. injecting drug use) is not collected as part of routine HIV testing, and thus proxy indicators were used to identify a cohort of individuals with evidence of drug use through data linkage (outlined below) and summarised in Appendix B.

Analysis was confined to a cohort of individuals who were either tested for HIV within a drug service or for whom there was information indicating drug use, obtained through linkage to the following three databases held at Public Health Scotland (PHS): Scottish Drugs Misuse Database (SDMD), Scottish Mortality Record 01 (SMR01, a national database of all hospital admissions to general/acute NHS specialities) and the hepatitis C (HCV) test database. We utilised all records within the SDMD which related to individuals who have been assessed to receive drug treatment in Scotland between 2006 and 2019 [14]. A subset of records from SMR01 relating to individuals who had been hospitalized for a drug‐related cause in Scotland during 1990–2019 was used based on a standard PHS definition [15] (Appendix C). The HCV test database contains a record of the vast majority of HCV‐positive and ‐negative tests (antibody, polymerase chain reaction (PCR) and antigen) in Scotland during 1997–2019 [16]; a subset of records from this database on individuals who had been tested in drug services was used.

In summary, individuals with evidence of drug use were identified as those who had an HIV test between 2000 and 2019, and had a record of at least one of the following: (i) a drug treatment assessment, (ii) a drug‐related hospital admission, or (iii) tested for HCV or HIV in a drug service. These individuals formed the ‘drug‐related cohort’ (referred to as such hereafter). Those who were included in the incidence analysis (referred to as ‘incidence cohort’ hereafter) were required to have at least two independent HIV tests: first tested HIV‐negative and have at least one subsequent independent HIV test >30 days later.

### Outcomes and exposures

The primary outcome was newly diagnosed HIV infection (referred to as incident HIV infection hereafter), defined as the date of the person's first HIV‐positive test result and confirmed with a PHS HIV diagnosis record as a new diagnosis (i.e. first ever positive antibody test). HIV diagnoses in Scotland are confirmed using HIV antigen/antibody tests and HIV‐1/HIV‐2 antibody assays and collated in a national HIV diagnosis database held at PHS [17, 18]. The key exposure variable was calendar year defined *a priori* as two pre‐outbreak periods (2000–2010 and 2011–2013), early outbreak (2014–2016) and ongoing outbreak (2017–2019). Other exposures included: sex (male/female), age group at first test (<35/35+ years), drug‐related hospital admission before first HIV test (yes/no), first test setting (categorized as: hospital/prison/drug service/primary care or other). Additionally, among those diagnosed with HIV, exposure variables derived from their record on the PHS HIV diagnosis database included: mode of acquisition category (via heterosexual intercourse/injecting drug use/sexual intercourse between gay, bisexual and other men who have sex with men/other and unknown) and confirmed to be associated with the HIV outbreak (yes/no).

### Statistical analysis

#### Characteristics of the drug‐related and incidence cohorts

Comparison of key covariates between those included and excluded from the incidence cohort was undertaken using χ^2^ tests. This was completed for all people identified in the drug‐related cohort and for those who were diagnosed with HIV.

#### HIV incidence

Incidence rates were computed using the person‐years (PY) method, and presented as the rate per 1000 PY [19, 20]. PY were calculated as time from an individual's first HIV‐negative test date to either their assumed infection date (defined as the midpoint between the first positive test and their most recent negative test prior to that) or their last negative test date (for those without a positive test). Incidence rates and 95% confidence intervals (CIs) were calculated assuming Poisson distribution across exposure covariates listed above.

Poisson regression models were fitted to estimate incidence rate ratios (IRRs) and adjusted incidence rate ratios (aIRRs) of HIV infection for those in the incidence cohort [19, 20]. Outbreak period was included as a time‐dependent covariate, where PYs and incident HIV diagnoses were attributed to the respective periods. IRR across outbreak periods were adjusted for gender, age at first test, drug‐related hospital admission before first HIV test and setting of first HIV test. Sensitivity analysis – as listed and described in Appendix D – was undertaken to assess whether the inclusion criteria for the drug‐related cohort influenced findings.

### Ethical and information governance approvals

Ethical approval was granted by the Glasgow Caledonian University Psychology, Social Work, and Allied Health Sciences Ethics Committee (HLS/PSWAHS/18/001). Relating to data linkage exercises, a Data Protection Impact Assessment (CAR18190329) was carried out and all data linkages were approved by the Public Benefit and Privacy Panel for Health and Social Care (1516‐0457).

## RESULTS

### Study population

The total number of individuals in the drug‐related cohort with at least one HIV test was 28 136; of those, 345 (1.2%) tested HIV‐positive (Tables 1 and 2). Of the 28 136 individuals, 59% (*n* = 16 661) were male, 51% (*n* = 14 238) were aged <35 years at first test and 35% (*n* = 9783) had a drug‐related hospital admission before their first HIV test. Nearly half (46%, *n* = 13 021) were first tested in either of the pre‐outbreak periods, and over half in the early (28%, *n* = 7806) or ongoing (26%, *n* = 7309) outbreak periods (Table 1).

**TABLE 1:** Characteristics of the drug‐related cohort tested for HIV in Glasgow during 2000–2019, according to whether were involved in incidence analysis.

Covariates	Drug‐related cohort (col%)	Involved in incidence analysis		*p*‐value^a^
		No (either positive on first HIV test or only one negative test) (col%)	Yes (first testing negative and ≥1 subsequent test) (col%)	
Total	28 136	14 652	13 484	‐
Period of first HIV test				
Pre‐outbreak (2000–2010)	8112 (29)	3732 (25)	4380 (32)	
Pre‐outbreak (2011–2013)	4909 (17)	2013 (14)	2896 (22)	<0.001
Early outbreak (2014–2016)	7806 (28)	3564 (24)	4242 (31)	
Ongoing outbreak (2017–2019)	7309 (26)	5343 (37)	1966 (15)	
Gender (115 NR)				
Female	11 360 (41)	6007 (41)	5353 (40)	
Male	16 661 (59)	8534 (59)	8127 (60)	0.006
Age group at first test (62 NR)				
<35 years	14 238 (51)	7464 (51)	6774 (50)	
35+ years	13 836 (49)	7126 (49)	6701 (50)	0.123
Drug‐related hospital admission before first HIV test				
Yes	9783 (35)	4434 (30)	5349 (40)	
No	18 353 (65)	10 218 (70)	8135 (60)	<0.001
First test setting				
Hospital	9863 (35)	4988 (34)	4875 (36)	
Prison	2856 (10)	1423 (10)	1433 (11)fG	
Drug service	5841 (21)	2836 (19)	3005 (22)	
Primary care/other settings^b^	9576 (34)	5405 (37)	4171 (31)	<0.001

Abbreviation: NR, not recorded.

^a^ Derived from χ^2^ test.

^b^ Includes antenatal, family planning, sexual health, occupational health, routine screen, other and unknown settings.

**TABLE 2:** Characteristics of drug‐related cohort who tested positive for HIV in Glasgow during 2000–2019, according to whether they were involved in incidence analysis.

Covariates	HIV positive individuals in drug‐related cohort (col%)	Involved in incidence analysis		*p*‐value^a^
		No (first tested positive) (col%)	Yes (first testing negative and ≥1 subsequent test) (col%)	
Total	345	201	144	‐
Period of first HIV test				
Pre‐outbreak (2000–2010)	144 (42)	77 (38)	67 (47)	
Pre‐outbreak (2011–2013)	73 (21)	38 (19)	35 (24)	
Early outbreak (2014–2016)	83 (24)	51 (26)	32 (22)	
Ongoing outbreak (2017–2019)	45 (13)	35 (17)	10 (7)	0.019
Period of positive HIV test				
Pre‐outbreak (2000–2010)	85 (25)	77 (38)	8 (6)	
Pre‐outbreak (2011–2013)	46 (13)	38 (19)	8 (6)	
Early outbreak (2014–2016)	115 (33)	51 (26)	64 (44)	
Ongoing outbreak (2017–2019)	99 (29)	35 (17)	64 (44)	<0.001
Gender				
Female	108 (31)	53 (26)	55 (38)	
Male	237 (69)	148 (74)	89 (62)	0.019
Age group at first test				
<35 years	168 (49)	82 (41)	86 (60)	
35+ years	177 (51)	119 (59)	58 (40)	0.001
Drug‐related hospital admission before first HIV test				
Yes	197 (57)	108 (54)	89 (62)	
No	148 (43)	93 (46)	55 (38)	0.135
First test setting				
Hospital	198 (57)	138 (69)	60 (42)	
Prison	44 (13)	19 (9)	25 (18)	
Drug service	54 (16)	24 (12)	30 (21)	
Primary care/other settings^b^	49 (14)	20 (10)	29 (20)	<0.001
Route of acquisition				
Heterosexual	46 (13)	35 (17)	11 (8)	
Injecting drug use	231 (67)	112 (56)	119 (82)	
Gay, bisexual and other men who have sex with men	64 (19)	52 (26)	12 (9)	
Other/unknown	4 (1)	1 (1)	2 (1)	<0.001
Associated with GGC HIV outbreak				
Yes	158 (46)	38 (19)	120 (83)	
No	187 (54)	163 (81)	24 (17)	<0.001

Abbreviation: NR, not recorded.

^a^ Derived from χ^2^ test.

^b^ Includes antenatal, family planning, sexual health, occupational health, routine screen, other and unknown settings.

For the incidence analysis, people who first tested HIV‐positive (0.7%, *n* = 201) and those who first tested negative but without a subsequent independent HIV test were excluded (52%, *n* = 14 652). A greater proportion of those excluded than included in the incidence analysis had been first tested in the ongoing outbreak period (2017–2019). This was expected as individuals required a follow‐up HIV test to be eligible for inclusion in the incidence cohort (Appendix F; Table 1). The incidence cohort included in the final analysis involved 13 484 people (48% of the total drug‐related cohort), relating to 48 309 HIV tests and 144 HIV incident diagnoses (Tables 1 and 2).

#### HIV‐diagnosed people

Among those included in the drug‐related cohort, 345 people were diagnosed with HIV in NHS GGC; 69% (*n* = 237) were male and 51% (*n* = 177) were aged over 35 years at their first HIV test. Over half were first tested pre‐outbreak (63%, *n* = 217) and diagnosed during the early and ongoing outbreak periods (62%; *n* = 214). The greatest proportion had injecting drug use as their route of acquisition (67%, *n* = 231) and just under half (46%, *n* = 158) were confirmed to be associated with the HIV outbreak (Table 2).

The majority (58%; *n* = 201) of HIV diagnoses tested positive on their first recorded test and were not included in the incidence cohort. Therefore, a total of 144 (42%) HIV‐positive individuals met the criterion for inclusion in the incidence cohort. A greater proportion of those excluded from the incidence cohort were diagnosed during the pre‐outbreak (2000–2010) period (38%, *n* = 77) as compared with those included in the incidence cohort (6%, *n* = 8) (*p* < 0.001). A greater proportion of those included (82%; *n* = 119) had injecting drug use listed as the main mode of acquisition category compared with those excluded (56%; *n* = 112; *p* < 0.001). Furthermore, among those included, 83% (*n* = 120) were confirmed HIV outbreak diagnoses, compared with 19% (*n* = 38) of those excluded (Table 2).

#### HIV incidence

**FIGURE 1:**
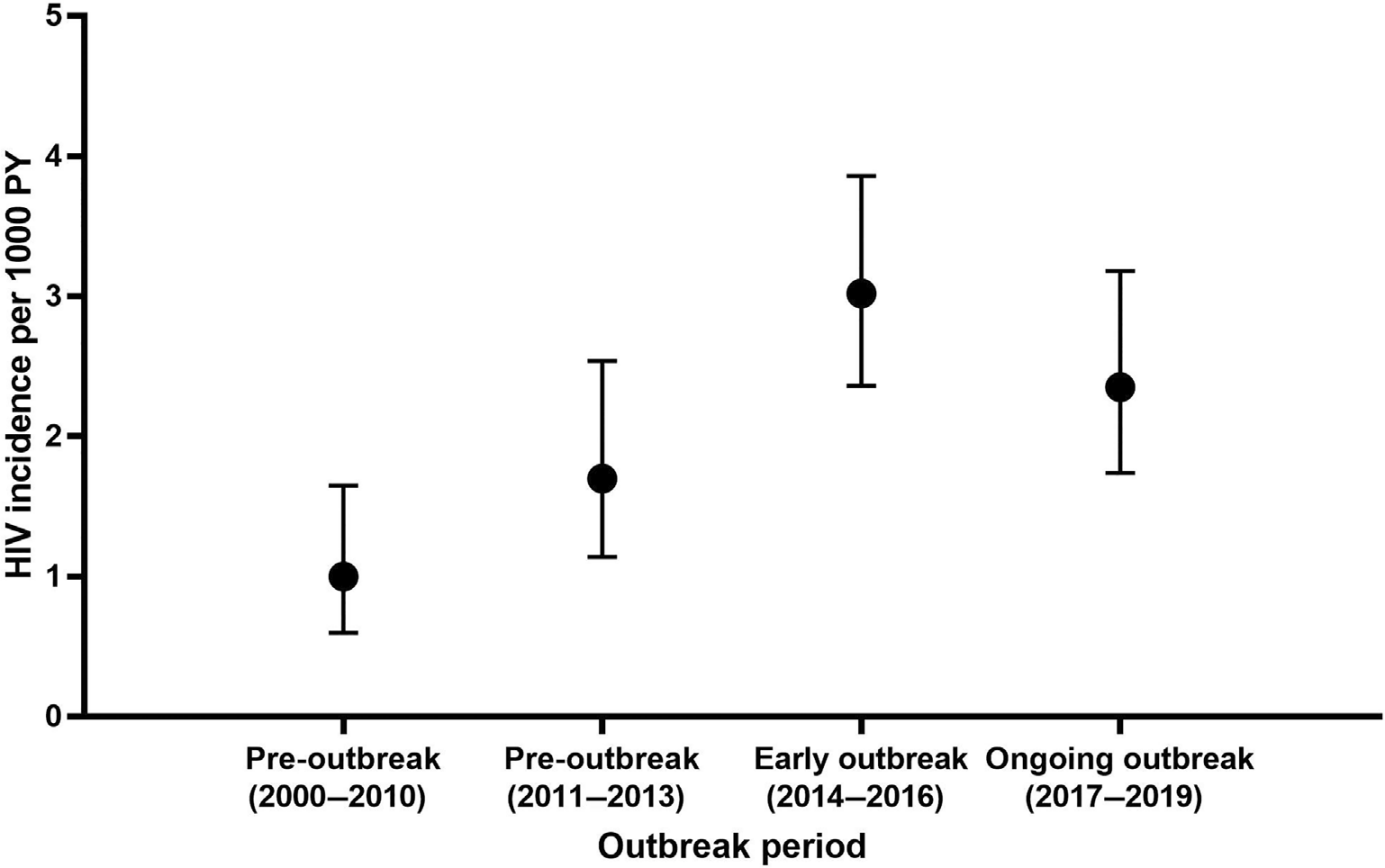
Estimated incidence of HIV among the incidence cohort (first testing negative and one or more subsequent tests) in Glasgow by outbreak period, 2000–2019. PY, person‐years.

A total of 144 individuals (1%) within the incidence cohort (*n* = 13 484) tested HIV‐positive. The estimated incidence rate was 2.12/1000 PY (95% CI: 1.80–2.49), based on a total of 67 906 PY (mean of 5 years per individual). The incidence rate observed rose from 1.00/1000 PY (95% CI: 0.60–1.65) in the pre‐outbreak period (2000–2010) to 1.70/1000 PY (95% CI: 1.14–2.54; *p* = 0.102) in the subsequent pre‐outbreak period (2011–2013), peaking in the early outbreak period (2014–2016) at 3.02/1000 PY (95% CI: 2.36–3.86; *p* < 0.001). Rates remained significantly elevated during the ongoing outbreak period (2017–2019) (2.35/1000 PY; 95% CI: 1.74–3.18; *p* = 0.004) compared with the pre‐outbreak (2000–2010) period (Table 3; Figure 1). In multivariate analysis, the risk of an incident HIV infection was significantly higher in both the early outbreak (2014–2016) (aIRR = 2.87, 95% CI: 1.62–5.09, *p* < 0.001) and ongoing outbreak (2017–2019) periods (aIRR = 2.12, 95% CI: 1.16–3.90, *p* = 0.015) than in the pre‐outbreak (2000–2010) period (Table 3, Figure 1). In addition, compared with the early HIV outbreak period (2014–2016), there was no significant change in the risk of an incident HIV infection in the ongoing outbreak period (2017–2019) (aIRR = 0.73, 95% CI: 0.50–1.11, *p* = 0.153) (Appendix G; Figure 1).

**TABLE 3:** Rate of, and factors associated with, HIV incidence in the incidence cohort (first testing negative and one or more subsequent test) in Glasgow, 2000–2019.

Covariates	*N*	HIV‐positive (row%)	Total person years	Incidence rate per 1000 person‐years (95% CI)	Univariate IRR (95% CI)	*p*‐value	Multivariate aIRR (95% CI)	*p*‐value
Total sample	13 484	144 (1.0)	67 906	2.12 (1.80–2.49)	‐	‐	‐	‐
Outbreak period^a^								
Pre‐outbreak (2000–2010)	4380	15 (0.3)	15 069	1.00 (0.60–1.65)	1		1	
Pre‐outbreak (2011–2013)	6610	24 (0.4)	14 079	1.70 (1.14–2.54)	1.71 (0.90–3.26)	0.102	1.72 (0.90–3.29)	0.099
Early outbreak (2014–2016)	10 050	63 (0.6)	20 873	3.02 (2.36–3.86)	3.03 (1.73–5.32)	<0.001	2.87 (1.62–5.09)	<0.001
Ongoing outbreak (2017–2019)	9761	42 (0.4)	17 885	2.35 (1.74–3.18)	2.36 (1.31–4.26)	0.004	2.12 (1.16–3.90)	0.015
Gender								
Female	5353	55 (1.0)	27 834	1.98 (1.51–2.57)	1		1	
Male	8127	89 (1.1)	40 065	2.22 (1.80–2.73)	1.12 (0.80–1.57)	0.495	0.88 (0.61–1.28)	0.515
Age group at first test								
<35 years	6774	86 (1.3)	39 193	2.19 (1.78–2.71)	1		1	
35+ years	6710	58 (0.9)	28 712	2.02 (1.56–2.61)	0.92 (0.66–1.28)	0.626	0.69 (0.49–1.00)	0.051
Drug‐related hospital admission before first HIV test								
No	8135	89 (1.6)	40 805	1.35 (1.03–1.76)	1		1	
Yes	5349	55 (0.7)	27 100	3.24 (2.67–4.04)	2.44 (1.74–3.41)	<0.001	2.34 (1.65–3.31)	<0.001
First test setting								
Primary care/other settings^b^	4171	29 (0.7)	22 292	1.30 (0.90–1.87)	1		1	
Drug service	3005	30 (1.0)	9764	3.07 (2.15–4.39)	2.36 (1.42–3.93)	0.001	2.04 (1.19–3.50)	0.009
Prison	1433	25 (1.7)	6924	3.61 (2.44–5.34)	2.78 (1.63–4.74)	<0.001	2.24 (1.26–3.99)	0.006
Hospital	4875	60 (1.2)	28 924	2.07 (1.61–2.67)	1.59 (1.02–2.48)	0.039	1.40 (0.89–2.20)	0.142

Abbreviations: CI, confidence interval; IRR, incidence rate ratio; aIRR, adjusted incidence rate ratio

^a^ Time‐dependent covariate.

^b^ Includes antenatal, family planning, sexual health, occupational health, routine screen, other and unknown settings.

## DISCUSSION

The overall aim of this study was to estimate temporal trends in HIV incidence in Glasgow over the course of the outbreak, and assess how the outbreak might have impacted these trends. To assess trends, we utilised a retrospective cohort study design that spanned a 19‐year period, including 6 years of data related to the HIV outbreak (2014–2019). A steady increase in HIV incidence was observed before the identification of the outbreak, which peaked in the early phase (2014–2016) at 3.02/1000 PY. In multivariate analysis, the incidence of HIV infection was approximately three‐fold higher in the early outbreak period (2014–2016) and two‐fold higher in the ongoing outbreak (2017–2019) period as compared with the pre‐outbreak rates (2000–2010).

Despite the importance of HIV incidence for measuring transmission dynamics, few settings that have experienced HIV outbreaks have used cohort studies to assess trends in HIV incidence [2]. At its peak, the HIV outbreak in Athens, Greece, resulted in an incidence rate of 78/1000 PY, and an outbreak in Tel Aviv, Israel, had an incidence rate of 20 per 1000 population. Our estimates are considerably lower, likely due to sample size and power [21]. Furthermore, we have generated incidence estimates for the whole of Glasgow due to limitations with the data. Our incidence rates would likely be much higher if focussed on data related to Glasgow city (the epicentre of the outbreak), and thus our estimates underestimate the overall burden of the outbreak. However, 83% (120/144) of HIV incident cases included in our analysis were attributed to the outbreak, and this represents 74% (120/168) of all HIV diagnoses associated with the outbreak from 2014 to 2019. Furthermore, the trends in incidence observed in this study are consistent with other epidemiological evidence, including a recently published mathematical modelling study which estimated incidence of HIV infection as part of an evaluation of the impact of the testing and treatment response among PWID in Glasgow [22], and a population‐based bio‐behavioural survey which estimated HIV prevalence among the population affected by the outbreak [3, 23].

We found elevated incidence rates in the ongoing outbreak period (2017–2019), which did not appear to reduce significantly when compared with the peak early outbreak incidence rates (2014–2016). This could be related to power to detect small significant changes in incidence [21]. However, when triangulated with other epidemiological evidence, these findings could suggest ongoing transmission associated with the outbreak before the COVID‐19 pandemic. A new transmission cluster was detected in 2019 [5], and 60% of new diagnoses in 2020 were recent infections (either classed through avidity testing or a recent negative HIV test), suggesting transmission in the previous year. Furthermore, the latest data on HIV prevalence in Glasgow relates to 2019, which suggests that HIV transmission had plateaued in Glasgow city centre but was increasing in areas surrounding Glasgow [24].

Low HIV testing rates among PWID were regarded as a key factor in the delayed detection and persistence of the outbreak [6]. Glasgow's public health response – involving the recommendation of opt‐out BBV testing in prisons and HIV testing on dried blood spot samples from drug services – yielded a doubling in testing coverage among PWID in Glasgow city centre, the epicentre of the outbreak [6]. However, the COVID‐19 pandemic has impacted the delivery of HIV prevention services, particularly HIV testing, in Glasgow and other settings that have experienced HIV outbreaks [25, 26]. The impact of the pandemic on HIV transmission associated with the Glasgow outbreak is currently unknown. However, a study from British Columbia, in Canada, found increased HIV transmission clusters associated with reduced access to health services due to the COVID pandemic, particularly among PWID, where clusters showed rapid growth compared with other at‐risk groups [27]. The continued monitoring of HIV incidence and HIV prevalence post‐COVID‐19 is fundamental to effectively inform public health responses to HIV in Glasgow, and in Scotland more widely.

The outbreak in Glasgow persisted for considerably longer than other outbreaks [2]. A potential explanation may be the unprecedented increase in powder cocaine injecting in Glasgow [3]. Cocaine is associated with more frequent injecting and, thus, a higher demand for clean injecting equipment [28]. NSP provisions may not have been sufficient to account for the increased frequency of injecting associated with cocaine use [29]. Although coverage of OAT in Glasgow was stable before the identification of the outbreak [3], OAT is specific for opioid dependence, and its effectiveness in reducing injecting risk behaviours and achieving improved HIV treatment outcomes is blunted in a scenario of a high prevalence of cocaine use [30]. Sexual transmission has elsewhere been found to be an important driver of HIV among PWID in the context of a high prevalence of cocaine use [31]. The harm reduction response in Glasgow initially focused on reducing injecting risk behaviours, and the contribution of sexual transmission may have been underestimated in the early stages of the outbreak. However, during the ongoing outbreak period, resource was directed to preventing sexual transmission (including the use of HIV pre‐exposure prophylaxis) [32]. Public health responses are urgently needed to address cocaine use in Glasgow. Trials of psychosocial interventions, such as contingency management, could be considered. A recent global systematic review found that contingency management initiatives (i.e. the provision of financial incentives) comprised the only intervention that has been associated with a reduction in stimulant use [33].

Responses to HIV outbreaks should include outreach, low threshold harm reduction (e.g. out‐of‐hours access or mobile harm reduction units) and the utilisation of peers, which have successfully engaged marginalised populations [2, 34]. Drug consumption rooms (DCRs) have been shown internationally to engage homeless individuals involved in public injecting (two key risk factors for HIV in Glasgow) [35]. The implementation of a DCR was proposed in response to the outbreak in 2016 [36] and, although there have been considerable obstacles to establishing a facility, one is expected to open in 2024. In addition, the clinical model implemented in response to the outbreak in Glasgow (involving intensive outreach and ART distribution alongside OAT) was a highly effective method for ensuring a high ART uptake among a marginalised homeless population, where 86% of individuals diagnosed achieved viral suppression by 2019 [5]. Modelling research demonstrated the effectiveness of this model in controlling HIV transmission when combined with increased HIV testing; HIV prevalence would have been three‐fold higher by the end of 2019 had this intervention not been put in place [22].

Several research designs have been used to estimate HIV incidence among PWID, including cohort studies [7, 20]; serial cross‐sectional surveys or routine HIV surveillance data combined with tests for recent HIV infection (i.e. antibody avidity testing indicating infection acquisition within the previous 3–4 months) [37, 38]; or modelling/statistical estimation based on changes in HIV prevalence [39]. Our methods highlight how linkage of routine laboratory testing data to other administrative data sources relating to drug‐related risk factors can be utilised to estimate HIV incidence. In comparison to other methods, this represents a relatively cost‐efficient and timely approach to gauge incidence and inform further monitoring efforts. Further, this approach (i.e. linkage of testing data to other administrative data to identify high‐risk populations) could also be applied to other populations (such as gay, bisexual and other men who have sex with men) or other infections (e.g. sexually transmitted infections). Ongoing measures and estimates of HIV incidence are fundamental to monitor progress towards HIV transmission elimination goals [11, 12].

### Strengths and limitations

Our study followed up a large sample (*n* = 13 484), equivalent to 67 906 PY of observation. Among those included in the incidence analysis, HIV diagnoses overwhelmingly represent those infected with HIV as part of the outbreak (83%). Due to limitations within the HIV testing data and the available information on locality, we could only assess HIV incidence at a health board level, which thus underestimated the overall burden of the outbreak. Furthermore, relating to pre‐outbreak incidence estimates, a substantial proportion (88%) of HIV diagnoses pre‐outbreak (during 2000–2013) did not meet the criteria for inclusion in incidence analyses. In addition, the majority of people (73%) who were first tested in the ongoing outbreak period (2017–2019) were also not included. Furthermore, to estimate the timing of infection, the mid‐point between a person's positive test and last negative result was adopted, which is commonly applied in the literature [20] but may have slightly overestimated the incidence rates pre‐outbreak. We have mitigated the limitations in our estimates by triangulating with other epidemiological evidence [3, 20, 22, 40].

Inclusion in the incidence cohort required two or more HIV tests. Low HIV testing rates among those at risk of infection, particularly in the early stages of the outbreak, will have impacted inclusion in the cohort (Appendix F) [6]. Furthermore, significant differences were observed between those who were included and excluded, and therefore estimates may not be representative of people who have undergone a single HIV test or those who have never been tested. Relating to data linkage, the laboratory HIV test data was linked to other healthcare data using CHI. This means that correspondence between HIV test records and individuals with a history of drug use will be subject to error (due to missing or incorrect CHI numbers). Furthermore, extensive sensitivity analyses were completed to assess any bias that may have arisen in the construction of the drug‐related cohort, and the trends observed remained robust (Appendix D).

### Conclusion

The public health response has helped to curb the increased incidence of HIV infection among people with a history of drug use in Glasgow, but further efforts will likely be needed to reduce it to levels observed prior to the outbreak. In the context of policy objectives to eliminate HIV transmission by 2030, addressing ongoing transmission among PWID should be a public health priority. We have demonstrated the utility of linking routine diagnostic test data in assessing and monitoring the incidence of HIV infection among individuals with a history of drug use. Such enhanced surveillance is important in identifying populations at higher risk, evaluating the impact of interventions and guiding strategies for prevention and treatment efforts to eliminate HIV transmission.

## AUTHOR CONTRIBUTIONS

KMAT, NEP, AM and SJM conceptualized the study design. KMAT completed the analysis under the supervision of NEP, AM and SJM, with statistical guidance from AY. SJS and RNG provided the HIV testing data used in this study. All authors contributed to the interpretation of the results. KMAT drafted the initial manuscript, and all authors reviewed, edited and approved the final version for submission.

## CONFLICT OF INTEREST STATEMENT

None to disclose.

## APPENDIX A SUMMARY OF PUBLIC HEALTH AND CONTROL MEASURES INTRODUCED IN RESPONSE TO THE GLASGOW HIV OUTBREAK

**Table:**

Response/control measures	Timeline and description
Needs assessment of people who inject drugs in public places in Glasgow city centre	2015–2016: Key recommendations were the establishment of a pilot drug consumption room (DCR) and co‐located heroin‐assisted treatment (HAT) service
Awareness raising and communication	2015: Development of posters, information leaflets for people who inject drugs (PWID) and frontline staff HIV training sessions with frontline staff Media releases Engagement group set up with PWID actively injecting in Glasgow city centre
Needle and syringe provision (NSP)	2016: New NSP sites to provide evening and weekend access introduced Lower dead space syringes introduced Third‐sector NSP outreach introduced 2019: Mobile harm reduction van; second van approved in August 2020
Opioid agonist therapy (OAT)	2015: Ensuring PWID diagnosed with HIV can access prompt OAT prescriptions 2019: Pilot HAT service embedded in homeless addictions heath service
HIV testing	2015: Systematic promotion of HIV tests on dried blood spot samples (>90%) from drug services Promotion of HIV testing in drug services and hospitals Pilot blood‐borne virus (BBV) testing in community pharmacy 2016: Opt‐out BBV testing in prisons 2018: Third‐sector point‐of‐care outreach testing Pilot incentivized HIV testing HIV pre‐exposure prophylaxis for individuals with a high risk of sexual transmission
HIV treatment	2016: Community dispensing of antiretroviral therapy (ART) alongside OAT BBV clinics in homeless addictions service (clinical management of cases and contact tracing) BBV outreach BBV clinical in reach to prisons (improve ART access for those in prison)

Source: NHS Greater Glasgow and Clyde Incident Management Team.

## APPENDIX C DEFINITION OF A DRUG‐RELATED HOSPITAL ADMISSION DIAGNOSIS USING INTERNATIONAL CLASSIFICATION OF DISEASE (ICD) 10 CODES

**Table:**

ICD‐10 code	Description
F11.0–F11.9	Mental and behavioural disorders due to: opioids
F12.0– F12.9	Mental and behavioural disorders due to: cannabinoids
F13.0– F.13.9	Mental and behavioural disorders due to: sedatives/hypnotics
F14.0–F14.9	Mental and behavioural disorders due to: cocaine
F15.0–F15.9	Mental and behavioural disorders due to: other stimulants
F16.0–F16.9	Mental and behavioural disorders due to: hallucinogens
F18.0–F18.9	Mental and behavioural disorders due to: volatile solvents
F19.0–F19.9	Mental and behavioural disorders due to: multiple/other drugs
T40.0	Poisoning by narcotics: opium
T40.1	Poisoning by narcotics: heroin
T40.3	Poisoning by narcotics: methadone
T40.5	Poisoning by narcotics: cocaine
T40.6	Poisoning by narcotics: unspecified narcotics
T40.7	Poisoning by narcotics: cannabis
T40.8	Poisoning by narcotics: LSD
T40.9	Poisoning by narcotics: unspecified hallucinogens
Codes below must also have any F11, F12‐16, F18 or F19 codes in the same admission	
T40.2	Poisoning by narcotics: other opioids
T40.4	Poisoning by narcotics: other synthetic narcotics
T42.3	Poisoning by antiepileptic, sedative‐hypnotic and antiparkinson drugs: barbituates
T42.4	Poisoning by antiepileptic, sedative‐hypnotic and antiparkinson drugs: benzodiazapines
T43.6	Poisoning by psychotropic drugs NEC: psychostimulants with abuse potential
T52	Toxic effect of organic solvents

## APPENDIX D SENSITIVITY ANALYSES TO ASSESS VALIDITY OF INCLUSION CRITERIA FOR DRUG‐RELATED COHORT

Sensitivity analysis was undertaken to assess whether the inclusion criteria for the drug‐related cohort influenced findings. In the main analysis, individuals formed the drug‐related cohort based on a drug‐related hospital admission, drug treatment assessment, or hepatitis C virus (HCV) or HIV test in a drug service before or at any point during the study period. In sensitivity analysis, inclusion criteria for the drug‐related cohort were restricted, as follows:

1. Drug‐treatment record, drug‐related hospital admission, or HCV test or HIV test within drug services before or within 1 year of first HIV test
2. Drug‐treatment record, drug‐related hospital admission, or HCV test or HIV test within drug services before or within 2 years of first HIV test
3. Drug‐treatment record, drug‐related hospital admission, or HCV test or HIV test within drug services before or within 5 years of first HIV test
4. Ever injected drug treatment record, injecting‐related hospital admission, or HCV or HIV test within drug service. *Ever injected* information was available on the Scottish Drugs Misuse Database (SDMD). Injecting‐related hospital admission was defined using ICD‐10 codes (Appendix E). Appropriate codes were selected relating to drugs known to be injected in Scotland and injecting‐related infections most commonly described in the literature.

In sensitivity analyses, 73% (*n* = 9824), 77% (*n* = 10 414) and 88% (*n* = 11 830) of the total incidence cohort had either a drug‐related hospital admission, drug treatment assessment, HCV or HIV test in the periods before/within 1 year, 2 years or 5 years of their first HIV test, respectively. In addition, 90% (*n* = 12 114) had either ever injected drugs recorded on their treatment assessment, an injecting‐related hospital admission, or been tested for HCV or HIV within a drug service. The risk of incident HIV infection in the early outbreak (2014–2016) and ongoing outbreak (2017–2019) periods remained elevated when compared with the pre‐outbreak (2000–2010) period in all sensitivity analyses (Appendix D.1).

### D.1. Sensitivity analyses: Assessing different definitions of the drug‐related cohort on incidence estimates among people with a history of drug use in Glasgow, 2000–2019

**Table:**

	*N* (col%)	HIV‐positive (row%)	Total person‐years	Incidence rate per 1000 person‐years (95% CI)	Univariate IRR (95% CI)	*p*‐value	Multivariate aIRR (95% CI)	*p*‐value
*(A) Drug‐treatment record, hospital admission, HCV or HIV test in drug service before or within 1 year of first HIV test* ^a^								
Total	9824	109 (1.1)	42 184	2.58 (2.14–3.12)	‐	‐	‐	‐
Outbreak period^a^, ^b^								
Pre‐outbreak (2000–2010)	2213	9 (0.4)	6775	1.33 (0.69–2.55)	1		1	
Pre‐outbreak (2011–2013)	4120	15 (0.3)	8085	1.86 (1.12–3.07)	1.39 (0.61–3.19)	0.248	1.60 (0.69–3.67)	0.264
Early outbreak (2014–2016)	7222	50 (0.7)	14 096	3.62 (2.75–4.76)	2.72 (1.34–5.53)	0.006	3.11 (1.51–6.40)	0.002
Ongoing outbreak (2017–2019)	7456	34 (0.5)	13 227	2.57 (1.84–3.59)	1.93 (0.92–4.03)	0.078	2.17 (1.01–4.64)	0.046
*(B) Drug‐treatment record, hospital admission, HCV or HIV test in drug service before or within 2 years of first HIV test* ^a^								
Total	10 414	115 (1.1)	44 707	2.57 (2.14–3.08)	‐	‐	‐	‐
Outbreak period^a^, ^b^								
Pre‐outbreak (2000–2010)	2401	10 (0.4)	7339	1.36 (0.73–2.53)	1		1	
Pre‐outbreak (2011–2013)	4362	15 (0.3)	8594	1.76 (1.05–2.89)	1.28 (0.58–2.85)	0.544	1.43 (0.64–3.19)	0.381
Early outbreak (2014–2016)	7635	55 (0.7)	14 863	3.70 (2.84–4.82)	2.72 (1.38–5.32)	0.004	3.00 (1.51–5.96)	0.002
Ongoing outbreak (2017–2019)	7836	35 (0.4)	13 909	2.51 (1.81–3.50)	1.85 (0.91–3.73)	0.087	1.99 (0.96–4.13)	0.062
*(C) Drug‐treatment record, hospital admission, HCV or HIV test in drug service before or within 5 years of first HIV test* ^a^								
Total	11 830	127 (1.1)	52 489	2.42 (2.03–2.88)	‐	‐	‐	‐
Outbreak period^a^, ^b^								
Pre‐outbreak (2000–2010)	2995	11 (0.4)	9040	1.22 (0.67–2.19)	1		1	
Pre‐outbreak (2011–2013)	5219	17 (0.3)	10 384	1.64 (1.02–2.63)	1.35 (0.63–2.87)	0.443	1.47 (0.68–3.14)	0.322
Early outbreak (2014–2016)	8766	60 (0.7)	17 338	3.36 (2.69–4.46)	2.84 (1.49–5.41)	0.001	3.04 (1.58–5.85)	0.001
Ongoing outbreak (2017–2019)	8726	39 (0.4)	15 727	2.48 (1.81–3.39)	2.04 (1.04–3.97)	0.037	2.12 (1.07–4.23)	0.032
*(D) Ever‐injected drug treatment record, injecting‐related hospital admission, HCV or HIV test within drug service* ^a^								
Total	12 114	139 (1.2)	63 014	2.21 (1.87–2.60)	‐	‐	‐	‐
Outbreak period^a^, ^b^								
Pre‐outbreak (2000–2010)	4380	15 (0.3)	13 985	1.00 (0.59–1.69)	1		1	
Pre‐outbreak (2011–2013)	6610	24 (0.4)	12 756	1.65 (1.07–2.52)	1.64 (0.84–3.23)	0.149	1.65 (0.84–3.25)	0.148
Early outbreak (2014–2016)	10 050	63 (0.6)	19 316	3.21 (2.50–4.11)	3.21 (1.79–5.72)	<0.001	3.05 (1.69–5.51)	<0.001
Ongoing outbreak (2017–2019)	9761	42 (0.4)	16 957	2.48 (1.83–3.35)	2.47 (1.35–4.53)	0.003	2.27 (1.22–4.24)	0.010

Abbreviations: CI, confidence interval; IRR, incidence rate ratio; aIRR, adjusted incidence rate ratio

^a^ Time‐dependent covariate.

^b^ Adjusted for gender, age, drug‐related hospital admission before first HIV test and first test setting.

## APPENDIX E DEFINITION OF AN INJECTING‐RELATED HOSPITAL ADMISSION DIAGNOSIS USING INTERNATIONAL CLASSIFICATION OF DISEASE (ICD) 10 CODES

**Table:**

ICD‐10 code	Description
F11	Mental and behavioural disorders due to: opioids
F13	Mental and behavioural disorders due to: sedatives/hypnotics
F14	Mental and behavioural disorders due to: cocaine
F15	Mental and behavioural disorders due to: other stimulants
F19	Mental and behavioural disorders due to: multiple/other drugs
T40.0	Poisoning by narcotics: opium
T40.1	Poisoning by narcotics: heroin
T40.3	Poisoning by narcotics: methadone
T40.5	Poisoning by narcotics: cocaine
T40.6	Poisoning by narcotics: unspecified narcotics
Codes below must also have codes F11, F13‐15, F19 in the same CIS	
T40.2	Poisoning by narcotics: other opioids
T42.2	Poisoning by antiepileptic, sedative‐hypnotic and antiparkinson drugs: benzodiazapines
I33	Acute and subacute infective endocarditis
L02	Cutaneous abscess, furuncle and carbuncle, unspecified
L03	Cellulitis
L08	Other local infections of skin and subcutaneous tissue
I80	Phlebitis and thrombophlebitis
A40	Septicaemia due to *Streptococcus*
A41	Other sepsis
A49.0, A49.1	Staphylococcal or streptococcal of unspecified site
M86	Osteomyelitis
A35	Other tetanus
M72.6	Necrotizing fasciitis
R22.2 – R22.9	Localized swelling, mass and lump on neck, trunk, upper limb, lower limb, multiple sites or unspecified
L97	Ulcer of lower limb, not elsewhere classified
L98.8	Other disorders of skin and subcutaneous tissue, not elsewhere classified: other specified disorders of skin and subcutaneous tissue
L98.4	Chronic ulcer of the skin, not elsewhere specified
M79.8, M79.9	Other or unspecified soft tissue disorder

## APPENDIX G RATE OF AND FACTORS ASSOCIATED WITH HIV INCIDENCE IN THE INCIDENCE COHORT (FIRST TESTING NEGATIVE AND ONE OR MORE SUBSEQUENT TESTS) IN GLASGOW, 2000–2019

**Table:**

	*N* (col%)	HIV‐positive (row%)	Total person‐years	Incidence rate per 1000 person‐years (95% CI)	Univariate IRR (95% CI)	*p*‐value	Multivariate aIRR (95% CI)	*p*‐value
Total sample	13 484	144 (1.0)	67 906	2.12 (1.80 to 2.49)	‐	‐	‐	‐
Outbreak period^a^, ^b^, ^c^								
Pre‐outbreak (2000–2010)	4380	15 (0.3)	15 069	1.00 (0.60–1.65)	0.33 (0.19–0.58)	<0.001	0.35 (0.19–0.62)	<0.001
Pre‐outbreak (2011–2013)	6610	24 (0.4)	14 079	1.70 (1.14–2.54)	0.56 (0.35–0.90)	0.017	0.59 (0.37–0.96)	0.035
Early outbreak (2014–2016)	10 050	63 (0.6)	20 873	3.02 (2.36–3.86)	1		1	
Ongoing outbreak (2017–2019)	9761	42 (0.4)	17 885	2.35 (1.74–3.18)	0.77 (0.53–1.15)	0.208	0.73 (0.50–1.10)	0.133

*Note*: For this analysis, early HIV outbreak (2014–2016) was used instead of pre‐outbreak 2000–2010 as the reference category for the outbreak period covariate in univariate and multivariate analysis.

Abbreviations: CI, confidence interval; IRR, incidence rate ratio; aIRR, adjusted incidence rate ratio

^a^ Time‐dependent covariate.

^b^ Includes antenatal, family planning, sexual health, occupational health, routine screen, other and unknown settings.

^c^ Adjusted for gender, age, drug‐related hospital admission before first HIV test and first test setting.
